# Value of Blood-Based microRNAs in the Diagnosis of Acute Myocardial Infarction: A Systematic Review and Meta-Analysis

**DOI:** 10.3389/fphys.2020.00691

**Published:** 2020-08-14

**Authors:** ChuanNan Zhai, Rui Li, Kai Hou, JingYi Chen, Mohammad Alzogool, YueCheng Hu, JingXia Zhang, YingYi Zhang, Le Wang, Rui Zhang, HongLiang Cong

**Affiliations:** ^1^School of Medicine, NanKai University, Tianjin, China; ^2^Department of Cardiology, Tianjin Chest Hospital, Tianjin, China; ^3^Tianjin GongAn Hospital, Tianjin, China

**Keywords:** miRNAs, acute myocardial infarction, diagnosis, biomarker, meta-analysis

## Abstract

**Background:** Recent studies have shown that blood-based miRNAs are dysregulated in patients with acute myocardial infarction (AMI) and are therefore a potential tool for the diagnosis of AMI. Therefore, this study summarized and evaluated studies focused on microRNAs as novel biomarkers for the diagnosis of AMI from the last ten years.

**Methods:** MEDLINE, the Cochrane Central database, and EMBASE were searched between January 2010 and December 2019. Studies that assessed the diagnostic accuracy of circulating microRNAs in AMI were chosen. The pooled sensitivity, specificity, positive likelihood ratio, negative likelihood ratio, diagnostic odds ratio, and area under the curve (AUC) were used to assess the test performance of miRNAs.

**Results:** A total of 58 studies that included 8,206 participants assessed the diagnostic accuracy of circulating miRNAs in AMI. The main results of the meta-analyses are as follows: (1) Total miRNAs: the overall pooled sensitivity and specificity were 0.82 (95% CI: 0.79–0.85) and 0.87 (95% CI: 0.84–0.90), respectively. The AUC value was 0.91 (95% CI: 0.88–0.93) in the overall summary receiver operator characteristic (SROC) curve. (2) The panel of two miRNAs: sensitivity: 0.88 (95% CI: 0.77–0.94), specificity: 0.84 (95% CI: 0.72–0.91), AUC: 0.92 (95% CI: 0.90–0.94). (3) The panel of three miRNAs: sensitivity: 0.91 (95% CI: 0.85–0.94), specificity: 0.87 (95% CI: 0.77–0.92), AUC: 0.92 (95% CI: 0.89–0.94). (4) Results by types of miRNAs: miRNA-1: sensitivity: 0.78 (95% CI: 0.71–0.84), specificity: 0.86 (95% CI: 0.77–0.91), AUC: 0.88 (95% CI: 0.85–0.90); miRNA-133a: sensitivity: 0.85 (95% CI: 0.69–0.94), specificity: 0.92 (95% CI: 0.61–0.99), AUC: 0.93 (95% CI: 0.91–0.95); miRNA-208b: sensitivity: 0.80 (95% CI: 0.69–0.88), specificity: 0.96 (95% CI: 0.77–0.99), AUC: 0.91 (95% CI: 0.88–0.93); miRNA-499: sensitivity: 0.85 (95% CI: 0.77–0.91), specificity: 0.95 (95% CI: 0.89–0.98), AUC: 0.96 (95% CI: 0.94–0.97).

**Conclusion:** miRNAs may be used as potential biomarkers for the detection of AMI. For single, stand-alone miRNAs, miRNA-499 may have better diagnostic accuracy compared to other miRNAs. We propose that a panel of multiple miRNAs with high sensitivity and specificity should be tested.

## Introduction

Although advanced clinical medications have recently been developed for the diagnosis and prevention of coronary heart disease (CAD), acute myocardial infarction (AMI), which includes ST-elevation myocardial infarction (STEMI) and non-STEMI (NSTEMI), is still considered a primary public health threat, with high morbidity and mortality worldwide (Moss et al., 1996; GBD 2013 Mortality and Causes of Death Collaborators, 2015). Acute-phase reaction during ischemic damage is a crucial pathogenesis of ischemia myocardial issue (Hoffmeister et al., 2003). Dependent on accurate recognition and diagnosis, early effective revascularization treatment is an important strategy to repair ischemic myocardium and can significantly reduce the mortality of AMI patients (Hung et al., 2013). Currently, the most widely used biomarkers of myocardial injury during clinical practice are cardiac troponin and creatine kinase-MB (CK-MB), which may provide effective benefits for patients with revascularization therapy (Dohi et al., 2015; Anand et al., 2019). However, the elevation of cardiac troponin may be involved in serious, non-cardiac disease such as neuromuscular disorders, severe sepsis, and chronic renal insufficiency (Lamb et al., 2006; Finsterer et al., 2007; Vallabhajosyula et al., 2017). High levels of cardiac troponin have also been detected in patients with heart failure (Myhre et al., 2018). Therefore, early diagnostic biomarkers and improvement of the accuracy of approaches for the early prediction of AMI are still warranted.

Potential novel genetic and molecular biomarkers are currently being explored (Lorenzano et al., 2019). MicroRNAs (miRNAs/miRs) are endogenous, non-coding RNAs ~19–25nt that play crucial post-regulatory roles in animals and plants by targeting mRNAs for translational or cleavage repression (Bartel, 2004). MiRNAs can inhibit or reduce target gene expression, subsequently affecting protein expression (Saxena et al., 2018). Thus, miRNAs play important regulatory roles in cell growth, development, and differentiation (Gabisonia et al., 2019). Further, miRNAs have been identified in extracellular fluid and can be extremely stable despite the presence of endogenous RNase (Chevillet et al., 2014). In recent years, a number of studies have reported that miRNAs are dysregulated in CAD, and that specific circulating miRNA signatures might be useful as biomarkers for the diagnosis of AMI and as therapeutic targets (Jakob et al., 2017). However, the results of previous studies were significantly different, potentially due to sample size, specimen types, and different detection technologies. Therefore, the purpose of this systematic review and meta-analysis was to summarize the diagnostic values of blood-based miRNA levels from published articles from the last 10 years and to appraise the accuracy of results to determine whether miRNAs may be used as novel biomarkers for the diagnosis of AMI.

## Materials and Methods

This study was conducted according to the Preferred Reporting Items for Systematic Reviews and Meta-analysis guidelines (Hutton et al., 2015; Moher et al., 2015). Two reviewers (CN Zhai, R Li) were independently involved with study selection, data extraction, and quality assessment.

### Study Selection

An electronic search of MEDLINE (including PubMed), the Cochrane Central database, and EMBASE was performed to identify relevant articles published between January 2010 and December 2019. The following medical subject heading terms were used: (“plasma” OR “serum” OR “circulating”) AND (“microRNA” OR “miRNAs” OR “miR^*^”) AND (“myocardial infarction” OR “AMI” OR “coronary heart disease” OR “coronary artery disease” OR “coronary syndrome” OR “ischemic heart disease”). No language restrictions were imposed. All relevant review articles were retrieved, and duplicates were removed by manually searching. Based on the title and abstract, manuscripts of interest were obtained for full-text review. Only full-text references were included.

### Inclusion and Exclusion Criteria

Inclusion and exclusion criteria were developed by the investigative team. The inclusion criteria were: (1) human studies, (2) studies related to circulating miRNAs levels and AMI, and (3) studies that contained enough data to evaluate the diagnostic value of miRNAs in AMI. Exclusion criteria were based on the following: (1) studies evaluating tissue miRNA or miRNA in other body fluids; (2) case reports, conference abstracts, and reviews; and (3) non-human studies.

### Data Extraction, Meta-Analysis, and Quality Assessment

Each manuscript was assessed independently by two researchers (CN Zhai and K Hou). Disagreements among reviewers were resolved by consensus. Data extracted included the following: authors, publication year, country, type of blood-based fluid (serum or plasma), characteristics of the study population (both case and control), study design (qRT-PCR detection method), whether miRNA screening was performed, number of miRNAs assessed, listing of the specific dysregulated miRNAs in AMI patients compared with controls, and outcome of statistical analyses including details of miRNA analysis, such as type of reference miRNA utilized. Studies reporting on single miRNA were included in the meta-analysis and were evaluated according to the Quality Assessment of Diagnostic Accuracy Studies 2 (QUADAS-2) checklist (Whiting et al., 2011), which is designed to assess the risk of bias and the applicability of studies of diagnostic accuracy. The following four key domains were included: patient selection, the index test, the reference standard, and flow and timing. Each was assessed with respect to the risk of bias, and the first three domains were assessed with respect to applicability.

### Statistical Analysis

Analysis was based on the accuracy of the identified miRNAs for diagnosing the presence of AMI, as determined using Receiver Operator Characteristic (ROC) curves via the Area Under the Curve (AUC) value, and sensitivity and specificity where available (Carter et al., 2016). We calculated the pooled sensitivity, specificity, positive likelihood ratio (PLR), negative likelihood ratio (NLR), and diagnostic odds ratio (DOR), generated the bivariate summary receiver operator characteristic (SROC) curve, and calculated the area under the curve (AUC) to assess the overall diagnostic accuracy of miRNAs in distinguishing AMI patients from controls. Forest plots were constructed using STATA (15.0 StataCorp LP, College, Station, TX, USA). Due to the presumed heterogeneity of studies, a random-effects model (DerSimonian–Laird method) was used (Mahid et al., 2006). The heterogeneity of included studies was assessed using *I*^2^, and the *P-*value was considered significant if *I*^2^ was >50% or *P* < 0.05. Subgroup analyses were performed to explore the potential source of heterogeneity as follows: (1) based on the type of blood sample (plasma or serum), (2) the method of qRT-PCR detection (SYBR Green or TaqMan), (3) the type of reference control used for normalization (RNU or Cel-miRNA), (4) sample size (Sample size ≥ 100 or Sample size <100), and (5) different populations (Caucasian or East Asian). To assess the publication bias of the included studies, we performed Deeks' test of funnel plot asymmetry (Deeks et al., 2005).

## Results

### Literature Search Results and Characteristics of the Included Studies

**Figure 1:**
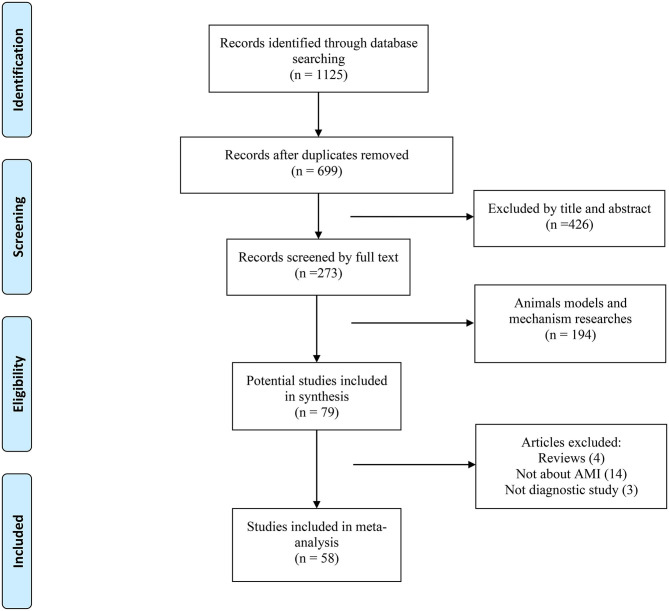
Flow diagram of the literature search process and study inclusion.

The PRISMA flow diagram of the literature search and inclusion of relevant studies are shown in Figure 1. Overall, 79 full-text articles were deemed relevant for a more detailed evaluation. Of these articles, 21 were excluded for the following reasons: review article (*n* = 4), not about AMI (*n* = 14), and not a diagnostic study (*n* = 3). Finally, 58 studies investigating plasma, serum, or peripheral venous blood miRNAs in the diagnosis of AMI were identified as eligible for inclusion in this systematic review (Adachi et al., 2010; Ai et al., 2010; Cheng et al., 2010; Corsten et al., 2010; D'Alessandra et al., 2010; Wang et al., 2010, 2011, 2013, 2014, 2016, 2019; Gidlöf et al., 2011, 2013; Meder et al., 2011; Zile et al., 2011; Devaux et al., 2012, 2015; Long et al., 2012a,b; Li C. et al., 2013; Li Y. Q. et al., 2013; Lu et al., 2013; Olivieri et al., 2013; He et al., 2014; Hsu et al., 2014; Huang et al., 2014; Li L. M. et al., 2014; Li Z. et al., 2014; Peng et al., 2014; Xiao et al., 2014; Białek et al., 2015; Chen et al., 2015; Gao et al., 2015; Han et al., 2015; Ji et al., 2015; Li et al., 2015; Liu et al., 2015, 2018; Yao et al., 2015; Zhang L. et al., 2015; Zhang R. et al., 2015; Zhao et al., 2015; Ke-Gang et al., 2016; Shalaby et al., 2016; Yang et al., 2016, 2017; Zhang et al., 2016, 2018; Zhu et al., 2016; Guo et al., 2017; Agiannitopoulos et al., 2018; Fawzy et al., 2018; Yi and An, 2018; Bukauskas et al., 2019; Li H. et al., 2019; Li P. et al., 2019; Xue et al., 2019a,b). These studies were performed in 12 countries; most of the subjects involved were East Asian, with Caucasian as the second most common population investigated. The major clinical characteristics of the included studies are shown in Table 1. In total, 8,206 patients were included in the study: 4,526 AMI patients and 3,680 healthy/non-AMI subjects (1,975 health controls, 1,705 non-AMI patients). Ten studies were relevant to the evaluation of patients with STEMI, three studies only included NSTEMI patients, and the other 45 articles included both types of myocardial infarction. The population demographics of our study are shown in Table 1. In total, 2,692 men and 1,834 women were included among AMI groups, and 2,066 men and 1,614 women were included in the control groups.

**Table 1:** Characteristics of studies included in the systematic review.

**References**	**Country**	**Specimen**	**Diseases**	**Case (*n*)**	**Control (*n*)**	**Age [mean** **±** **s.d. /(range)]**		**Gender (male: female)**	
						**Case**	**Control**	**Case**	**Control**
Corsten et al. (2010)	Netherlands	Plasma	STEMI vs. NC	32	36	62 ± 13	62 ± 13	24/12	23/13
Adachi et al. (2010)	Japan	Plasma	AMI vs. NC	9	10	66.8 ± 9.28	41.5 ± 8.0	6/3	5/5
Ai et al. (2010)	China	Plasma	AMI vs. NC	93	66	58.2 ± 10.2	55.1 ± 9.6	67/26	39/27
Wang et al. (2010)	China	Plasma	AMI vs. non-AMI	33	33	63.5 ± 10.1	64.3 ± 7.6	23/10	22/11
Cheng et al. (2010)	China	Serum	AMI vs. NC	31	20	NA (45-71)	NA	18/13	NA
D'Alessandra et al. (2010)	Italy	Plasma	STEMI vs. NC	33	17	57.9 ± 8.6	46.1 ± 13.9	31/2	13/4
Gidlöf et al. (2011)	Sweden	Plasma	STEMI vs. NC	25	11	64.56 ± 2.7	65.09 ± 3.51	20/5	7/4
Meder et al. (2011)	Germany	Blood	AMI vs. NC	20	20	59.3 ± 14	63.3 ± 14.8	16/4	14/6
Wang et al. (2011)	China	Plasma	AMI vs. NC	51	28	60.06 ± 11.53	57.86 ± 10.36	43/8	19/9
Zile et al. (2011)	USA	Plasma	AMI vs. NC	12	12	58 ± 3	61 ± 2	9/3	5/7
Devaux et al. (2012)	Netherlands	Plasma	STEMI & NSTEMI vs. NC	510	87	62 (0.32–91)	53 (40–60)	303/94	87/0
Long et al. (2012a)	China	Plasma	AMI vs. NC	17	25	53 ± 12.5	51 ± 12.3	13/4	18/7
Long et al. (2012b)	China	Plasma	AMI vs. NC	18	30	55 ± 11.4	50 ± 12.3	13/5	17/13
Li Y. Q. et al. (2013)	China	Plasma	AMI vs. NC	67	32	63.84 ± 11.17	61.75 ± 9.58	52/15	22/10
Li C. et al. (2013)	China	Serum	AMI vs. NC	117	100	62.7 ± 11.4	65.3 ± 9.98	20/97	19/81
Lu et al. (2013)	China	Plasma	AMI vs. non-AMI	40	15	66.8 ± 11.1	54.3 ± 17.5	30/10	6/9
Gidlöf et al. (2013)	Sweden	Plasma	AMI vs. non-AMI	319	88	NA	NA	NA	NA
Olivieri et al. (2013)	Italy	Plasma	NSTEMI vs. NC	92	99	82.6 ± 6.9	79.5 ± 5.4	39/53	40/59
Wang et al. (2013)	China	Plasma	AMI vs. NC	13	27	NA	NA	NA	NA
Li L. M. et al. (2014)	China	Plasma	AMI vs. NC	56	28	63.95 ± 11.34	60.50 ± 9.10	44/12	20/8
Li Z. et al. (2014)	China	Plasma	AMI vs. NC	27	31	54.15 ± 11.34	51.21 ± 12.25	20/7	16/15
Huang et al. (2014)	China	Plasma	AMI vs. NC	150	150	60.93 ± 12.86	60.66 ± 9.94	122/28	123/27
Hsu et al. (2014)	Tai Wan	Serum	STEMI vs. NC	31	31	59.0 ± 11.5	53.7 ± 14.8	29/2	29/2
He et al. (2014)	China	Plasma	AMI vs. non-AMI	359	30	58 ± 14	57 ± 10	301/58	21/9
Peng et al. (2014)	China	Plasma	AMI vs. non-AMI	76	110	64.6 (46–88)	60 (52–81)	43/33	61/49
Wang et al. (2014)	China	Plasma	AMI vs. NC	17	28	52 ± 11	58 ± 11	12/5	12/16
Xiao et al. (2014)	China	Serum	AMI vs. NC	NA	NA	NA	NA	NA	NA
Białek et al. (2015)	Poland	Plasma	STEMI vs. non-AMI	19	20	58 (55–65)	63 (58–74)	15/4	8/12
Chen et al. (2015)	China	blood	AMI vs. non-AMI	53	50	68.8 ± 7.3	68.3 ± 6.5	44/9	39/11
Devaux et al. (2015)	Luxembourg	Plasma	AMI vs. non-AMI	224	931	72 (61–80)	61 (49–74)	158/66	610/321
Gao et al. (2015)	China	Plasma	STEMI vs. non-AMI	35	160	NA	NA	NA	NA
Ji et al. (2015)	China	Serum	AMI vs. NC	98	23	64 ± 13.8	63.6 ± 12.2	82/16	15/8
Han et al. (2015)	China	Plasma	AMI vs. non-AMI	42	42	69.2 ± 7.4	67.4 ± 5.4	34/8	33/9
Li et al. (2015)	China	Plasma	STEMI & NSTEMI Vs. NC	87	87	56.93 ± 9.17	57.28 ± 10.82	64/23	62/25
Liu et al. (2015)	China	Plasma	AMI vs. NC	70	72	64.2 ± 11.2	62.3 ± 10.3	34/36	35/37
Yao et al. (2015)	China	Plasma	AMI vs. NC	50	39	63.2 ± 11.4	62.7 ± 10.5	33/17	27/12
Zhang L. et al. (2015)	China	Plasma	AMI vs. non-AMI	142	85	64.86 ± 12.84	66.45 ± 10.61	102/40	59/26
Zhang R. et al. (2015)	China	Plasma	STEMI & NSTEMI	110	110	57.74 ± 12.03	58.28 ± 11.32	87/23	83/27
Zhao et al. (2015)	China	Serum	AMI vs. NC	59	60	60.1 ± 11.3	61.9 ± 12.1	34/25	30/30
Ke-Gang et al. (2016)	China	Plasma	AMI vs. non-AMI	233	79	63 (20-91)	69 (35-70)	163/70	46/33
Wang et al. (2016)	China	Plasma	AMI vs. NC	32	36	55.62 ± 9.17	58.57 ± 11.54	18/14	24/12
Yang et al. (2016)	China	Plasma	AMI vs. NC	54	30	NA	NA	NA	NA
Zhang et al. (2016)	China	Plasma	AMI vs. NC	17	10	59.7 ± 8.4	56.2 ± 5.5	12/5	5/5
Zhu et al. (2016)	China	Plasma	STEMI vs. NC	60	60	64.1 ± 10.3	62.1 ± 11.2	46/14	45/15
Shalaby et al. (2016)	Egypt	Serum	NSTEMI vs. NC	48	25	54.3 ± 8.3	49.2 ± 10.2	32/16	17/8
Fawzy et al. (2018)	Egypt	Serum	STEMI vs. NC	110	121	NA	NA	61/49	63/58
Guo et al. (2017)	China	Plasma	AMI vs. NC	90	45	35.2 ± 5.6	36.2 ± 5.2	48/42	25/20
Yang et al. (2017)	China	Serum	AMI vs. NC	76	30	63.6 ± 11.7	62.3 ± 10.5	48/28	19/11
Agiannitopoulos et al. (2018)	Greece	Plasma	AMI vs. NC	50	50	62.12 ± 10.99	59.30 ± 9.82	38/12	33/17
Liu et al. (2018)	China	Plasma	NSTEMI vs. NC	145	30	67	65	56:89	15/15
Wang et al. (2019)	China	Plasma	AMI vs. NC	66	70	61.84 ± 1.71	61.90 ± 3.69	30/36	26/44
Yi and An (2018)	China	Plasma	AMI vs. NC	30	30	61.35 ± 8.65	57.64 ± 5.91	NA	NA
Zhang et al. (2018)	China	Plasma	STEMI vs. NC	80	60	62.83 ± 7.52	63.15 ± 7.32	46/34	35/25
Bukauskas et al. (2019)	Lithuania	Serum	STEMI vs. NC	62	26	64 ± 12	42 ± 13	46/16	20/6
Li H. et al. (2019)	China	Plasma	AMI vs. NC	35	55	60.86 ± 11.25	56.36 ± 12.36	15/20	27/28
Li P. et al. (2019)	China	Serum	AMI vs. non-AMI	41	32	62.95 ± 11.04	63.16 ± 10.63	30/11	19/13
Xue et al. (2019a)	China	Plasma	STEMI & NSTEMI	29	21	68.0 ± 10.4	58.5 ± 14.3	23/6	16/5
Xue et al. (2019b)	China	Plasma	STEMI & NSTEMI	31	27	61.1 ± 10.0	60.1 ± 12.2	25/6	19/8

*STEMI, ST-elevation myocardial infarction; AMI, acute myocardial infarction; NSTEMI, non ST-elevation myocardial infarction; NC, normal control*.

### Identification of Dysregulated miRNAs in the Included Studies

All included studies used Quantitative reverse transcription polymerase chain reaction (qRT-PCR) to detect the expression levels of miRNAs. A summary of all study methods is provided in Table 2. Seven studies performed miRNA screening to compare blood-based miRNAs between AMI patients and control groups (Adachi et al., 2010; D'Alessandra et al., 2010; Meder et al., 2011; Li C. et al., 2013; Hsu et al., 2014; Huang et al., 2014; Li et al., 2015). Fifty-one articles identified miRNAs based on their own previous studies or based on the literature (Ai et al., 2010; Cheng et al., 2010; Corsten et al., 2010; Wang et al., 2010, 2011, 2013, 2014, 2016, 2019; Gidlöf et al., 2011, 2013; Zile et al., 2011; Devaux et al., 2012, 2015; Long et al., 2012a,b; Li Y. Q. et al., 2013; Lu et al., 2013; Olivieri et al., 2013; He et al., 2014; Li L. M. et al., 2014; Li Z. et al., 2014; Peng et al., 2014; Xiao et al., 2014; Białek et al., 2015; Chen et al., 2015; Gao et al., 2015; Han et al., 2015; Ji et al., 2015; Liu et al., 2015, 2018; Yao et al., 2015; Zhang L. et al., 2015; Zhang R. et al., 2015; Ke-Gang et al., 2016; Shalaby et al., 2016; Yang et al., 2016, 2017; Zhang et al., 2016, 2018; Zhu et al., 2016; Guo et al., 2017; Agiannitopoulos et al., 2018; Fawzy et al., 2018; Yi and An, 2018; Bukauskas et al., 2019; Li H. et al., 2019; Li P. et al., 2019; Xue et al., 2019a,b). The expression of 50 miRNAs were identified as either significantly higher or lower expression in AMI cases; specific details were shown in Table 3. Thirty-three miRNAs were upregulated (miRNA-1, miRNA-17-5p, miRNA-19b-3p, miRNA-21, miRNA-23b, miRNA-26a-1, miRNA-30a, miRNA-122-5p, miRNA-124, miRNA-126, miRNA-133a/b, miRNA-134, miRNA-145-3p, miRNA-146a, miRNA-150, miRNA-181a, miRNA-186, miRNA-195, miRNA-199a-1, miRNA-208a/b, miRNA-210, miRNA-223, miRNA-302b, miRNA-328, miRNA-361-5p, miRNA-423-5p, miRNA-486, miRNA-494, miRNA-497, miRNA-499, miRNA-663b, miRNA-1291, and miRNA-1303) (Adachi et al., 2010; Ai et al., 2010; Cheng et al., 2010; Corsten et al., 2010; D'Alessandra et al., 2010; Wang et al., 2010, 2011, 2013, 2014, 2016, 2019; Gidlöf et al., 2011, 2013; Meder et al., 2011; Zile et al., 2011; Devaux et al., 2012, 2015; Long et al., 2012a,b; Li C. et al., 2013; Li Y. Q. et al., 2013; Olivieri et al., 2013; He et al., 2014; Hsu et al., 2014; Li L. M. et al., 2014; Li Z. et al., 2014; Xiao et al., 2014; Białek et al., 2015; Chen et al., 2015; Han et al., 2015; Ji et al., 2015; Li et al., 2015; Liu et al., 2015, 2018; Yao et al., 2015; Zhang L. et al., 2015; Zhang R. et al., 2015; Ke-Gang et al., 2016; Shalaby et al., 2016; Zhang et al., 2016, 2018; Guo et al., 2017; Yang et al., 2017; Agiannitopoulos et al., 2018; Fawzy et al., 2018; Li P. et al., 2019; Xue et al., 2019a,b), and 17 miRNAs were downregulated (miRNA-22-5p, miRNA-23a-3p, miRNA-26a, miRNA-30d-5p, miRNA-99a, miRNA-125b, miRNA-126-3p, miRNA-132-5p, miRNA-145, miRNA-146a-5p, miRNA-191, miRNA-214, miRNA-320b, miRNA-375, miRNA-379, miRNA-519-5p, and miRNA-let-7d) (D'Alessandra et al., 2010; Meder et al., 2011; Long et al., 2012b; Lu et al., 2013; Hsu et al., 2014; Huang et al., 2014; Wang et al., 2014, 2019; Gao et al., 2015; Li et al., 2015; Yang et al., 2016; Yi and An, 2018; Bukauskas et al., 2019; Li H. et al., 2019). Thirteen upregulated miRNAs (miRNA-1, miRNA-19b-3p, miRNA-21, miRNA-122-5p, miRNA-126, miRNA-133a/b, miRNA-134, miRNA-150, miRNA-186, miRNA-208a/b, miRNA-486, miRNA-499, and miRNA-663b) (Adachi et al., 2010; Corsten et al., 2010; D'Alessandra et al., 2010; Wang et al., 2010, 2011, 2013, 2016, 2019; Gidlöf et al., 2011, 2013; Devaux et al., 2012, 2015; Li Y. Q. et al., 2013; Olivieri et al., 2013; He et al., 2014; Hsu et al., 2014; Peng et al., 2014; Xiao et al., 2014; Białek et al., 2015; Chen et al., 2015; Han et al., 2015; Ji et al., 2015; Li et al., 2015; Liu et al., 2015, 2018; Zhang L. et al., 2015; Zhang R. et al., 2015; Ke-Gang et al., 2016; Shalaby et al., 2016; Agiannitopoulos et al., 2018; Fawzy et al., 2018; Li H. et al., 2019; Li P. et al., 2019) and three downregulated miRNAs (miRNA-26a, miRNA-191, and miRNA-375) (D'Alessandra et al., 2010; Hsu et al., 2014; Li et al., 2015; Wang et al., 2019) were identified by more than one study. Among these articles, which included 22 original studies, miRNA-499 was the most frequently identified dysregulated miRNA in AMI patients (Adachi et al., 2010; Corsten et al., 2010; D'Alessandra et al., 2010; Wang et al., 2010; Gidlöf et al., 2011, 2013; Devaux et al., 2012, 2015; Li C. et al., 2013; Li Y. Q. et al., 2013; Olivieri et al., 2013; Xiao et al., 2014; Chen et al., 2015; Liu et al., 2015, 2018; Zhang L. et al., 2015; Zhang R. et al., 2015; Shalaby et al., 2016; Agiannitopoulos et al., 2018; Fawzy et al., 2018; Li P. et al., 2019). 16 studies focused on the miRNA-208 family (miRNA-208a/b) (Corsten et al., 2010; Wang et al., 2010; Gidlöf et al., 2011, 2013; Devaux et al., 2012, 2015; Li C. et al., 2013; Li Y. Q. et al., 2013; Xiao et al., 2014; Białek et al., 2015; Han et al., 2015; Li et al., 2015; Liu et al., 2015, 2018; Agiannitopoulos et al., 2018; Li P. et al., 2019), 14 studies focused on miRNA-1 (Ai et al., 2010; Cheng et al., 2010; Corsten et al., 2010; D'Alessandra et al., 2010; Wang et al., 2010; Gidlöf et al., 2011, 2013; Long et al., 2012a; Li C. et al., 2013; Li Y. Q. et al., 2013; Olivieri et al., 2013; Li L. M. et al., 2014; Liu et al., 2015, 2018), and 14 studies focused on the miRNA-133 family (miRNA-133a/b) (Corsten et al., 2010; D'Alessandra et al., 2010; Wang et al., 2010, 2011, 2013; Gidlöf et al., 2011; Li Y. Q. et al., 2013; Olivieri et al., 2013; Peng et al., 2014; Ji et al., 2015; Ke-Gang et al., 2016; Liu et al., 2018). Additionally, four studies identified a panel of two miRNAs (Hsu et al., 2014; Zhang R. et al., 2015; Shalaby et al., 2016; Wang et al., 2019), six studies identified a panel of three miRNAs (Long et al., 2012b; Wang et al., 2014, 2016; Li H. et al., 2019; Xue et al., 2019a,b), and one study identified a panel of more than four miRNAs that were elevated in AMI (Li C. et al., 2013).

**Table 2:** Study methods and corresponding dysregulated miRNAs identified.

**References**	**qRT-PCR detection method**	**Reference control**	**miRNA screening performed**	**Dysregulated miRNAs^a^ (n)**	**Significantly dysregulated miRNAs on validation (*n*)**	**AMI definition**	**Time of blood sampling**
Corsten et al. (2010)	SYBR	n/a	N	6	2	ST segment elevation; increase CK and TnI	within 12 h of chest pain onset
Adachi et al. (2010)	TaqMan	n/a	Y	3	1	NA	Within 48 h after onset of chest pain
Ai et al. (2010)	SYBR	RNU6	N	2	1	Ischemic symptoms; increase cTnI and CK-MB; ST segment elevation or depression; pathological Q wave	NA
Wang et al. (2010)	TaqMan	Cel-miR-39	N	40	4	Ischemia symptom; increase cTnI; ST segment change; coronary angiography	Within 12 h after admission
Cheng et al. (2010)	n/a	n/a	N	1	1	Ischemic chest pain; increase CK-MB; ST segment elevation	Within 24 h after AMI
D'Alessandra et al. (2010)	TaqMan	n/a	Y	48	6	NA	Within 12 h after the onset of symptoms
Gidlöf et al. (2011)	SYBR	miR-16	N	5	4	ST-segment elevation; coronary angiography	Within 24 h of the onset of ischemic symptoms
Meder et al. (2011)	n/a	RNU6	Y	40	20	ESC/AHA guidelines	NA
Wang et al. (2011)	SYBR	RNU6	N	2	2	Chest pain lasting >20 min; increase CK-MB and cTnI; ST segment and T- wave changes; pathological Q wave	Within 24 h after the onset of syndromes
Zile et al. (2011)	TaqMan	RNU6	N	6	5	AHA/ACC guidelines	Within 24 h after admission
Devaux et al. (2012)	TaqMan	n/a	N	2	2	ST segment elevation or depression; increase CK and TnI; coronary angiography	With acute and ongoing chest pain for 12 h
Long et al. (2012a)	SYBR	RNU6	N	2	2	Ischemic symptom; increase cTnI and CK-MB; ST segment elevation or depression; pathological Q wave;	Within 4 h after onset of symptom
Long et al. (2012b)	SYBR	RNU6	N	3	3	Ischemic symptom; increase cTnI and CK-MB; ST segment elevation or depression; pathological Q wave;	Within 4 h after onset of symptom
Li Y. Q. et al. (2013)	SYBR	Cel-miR-39	N	4	4	Chest pain lasting >30 min; increase CK-MB and cTnI; ST segment elevation or depression; pathological Q wave	Within 12 h of the onset of symptoms
Li C. et al. (2013)	TaqMan	n/a	Y	21	6	Chest pain; ST segment elevation or depression; increase cTnI and CK-MB; pathological Q wave	Within 2h after hospitalization
Lu et al. (2013)	TaqMan	RNU6	N	1	1	Typical chest pain; increase cTnI; coronary angiography	The next morning after admission
Gidlöf et al. (2013)	SYBR	miR-17	N	3	2	STEMI: ECG criteria; NSTEMI: increase troponin and clinical symptoms	71% were taken within 24 h, 82% within 48 h and 93% within 72 h after onset of chest pain
Olivieri et al. (2013)	TaqMan	miR-17, cel-miR-39	N	6	5	Ischemic symptom; ST segment elevation or depression >1 mm/negative T wave/new onset LVBB; increase cTnT	Immediately after hospitalization
Wang et al. (2013)	SYBR	RNU6	N	1	1	Acute ischemic chest pain within 24 h; ECG changes; increase cTnI	Immediately after admission
Li L. M. et al. (2014)	SYBR	Cel-miR-39	N	1	1	Increased cTnT or CK-MB; chest pain lasting for >30 min; pathological Q waves/ST-segment changes	Within 12 h after onset of chest pain
Li Z. et al. (2014)	SYBR	RNU6	N	1	1	Ischemic symptoms; increase cTnI and CK-MB; ST segment elevation or depression; pathological Q wave	Within 4 h after onset of symptom
Huang et al. (2014)	n/a	Cel-miR-39	Y	77	2	Chest paining lasting >20 min; ST segment changes; pathological Q wave; increase cardiac biomarkers	NA
Hsu et al. (2014)	SYBR	n/a	Y	25	5	ACC/AHA guideline	NA
He et al. (2014)	SYBR	n/a	N	2	2	Ischemic symptoms; increase cTn and CK; ST segment change; pathological Q wave	6 h after the onset of symptoms
Peng et al. (2014)	TaqMan	miR-16	N	3	3	STEMI: ST segment elevation; NSTEMI: ischemic symptom and increase cTnI	Within 3 h after admission
Wang et al. (2014)	SYBR	RNU6	N	3	3	Acute ischemic-type chest pain; ECG changes; increase cTnI	Immediately after admission
Xiao et al. (2014)	TaqMan	Cel-miR-39	N	2	2	NA	NA
Białek et al. (2015)	TaqMan	HY3	N	1	1	Chest pain; ST segment elevation; coronary angiography	NA
Chen et al. (2015)	TaqMan	RNU6	N	1	1	Biochemical markers; acute ischemic-type chest pain; ECG changes; coronary angiography	Immediately after admission
Devaux et al. (2015)	SYBR	n/a	N	6	3	ACC/AHA guideline	With acute chest pain for 12 h
Gao et al. (2015)	SYBR	Cel-miR-39	N	1	1	ACC/AHA guidelines	Immediately after hospitalization
Ji et al. (2015)	SYBR	miR-16	N	3	3	Ischemia symptoms; ST segment elevation; increase cTnI and CK-MB	Immediately after hospitalization
Han et al. (2015)	TaqMan	RNU6	N	1	1	ESC/AHA/ACC guidelines	Within 12 h after the symptom onset
Li et al. (2015)	TaqMan	RNU6	Y	28	3	Ischemia symptoms; ST segment abnormality; pathological Q wave; increase cTnI and CK-MB	Within 4 h after onset of symptoms
Liu et al. (2015)	TaqMan	Cel-miR-39	N	3	3	Increased cTnT or CK-MB; chest pain lasting for >30 min; pathological Q waves/ST-segment changes	Within 2 h after the onset of symptom
Yao et al. (2015)	SYBR	RNU6	N	1	1	Ischemic symptoms; increase cTnI and CK-MB; ST segment changes and pathological Q wave	Within 4 h after the onset of symptoms
Zhang L. et al. (2015)	TaqMan	RNU6	N	1	1	Ischemic-type chest pain; increase cTnI; ECG change; coronary angiography	Within 12 h after the onset of acute chest pain
Zhang R. et al. (2015)	TaqMan	RNU6	N	2	2	Acute ischemic chest pain; abnormal ECG; increase cTnI and CK	Within 24 h after admission
Zhao et al. (2015)	SYBR	Cel-miR-39	N	1	1	ESC/ACC guidelines	Within 24 h after hospitalization
Ke-Gang et al. (2016)	TaqMan	Cel-miR-39	N	1	1	ESC Guidelines	Immediately after hospitalization
Wang et al. (2016)	SYBR	Cel-miR-39	N	3	3	ESC/AHA/ACC guidelines	Within 12 h after the onset of chest pain
Yang et al. (2016)	SYBR	RNU6	N	1	1	Coronary angiography	NA
Zhang et al. (2016)	TaqMan	Cel-miR-39	N	1	1	ESC/ACC guidelines	Immediately after admission
Zhu et al. (2016)	TaqMan	RNU6	N	1	1	Ischemic chest pain lasting>30 min; increase cTnI; new ST segment elevation	Immediately after admission
Shalaby et al. (2016)	SYBR	RNU6	N	2	2	Ischemic symptom; no ST segment elevation; increase cTnI; coronary angiography	Within 24 h of onset of chest pain
Fawzy et al. (2018)	TaqMan	RNU6	N	1	1	Ischemic symptoms; ECG changes; coronary angiography	Within the first 12 h of the chest pain
Guo et al. (2017)	n/a	GAPDH	N	1	1	Guideline for AMI by Chinese Medicine Academy	Within 12 h of chest pain onset
Yang et al. (2017)	SYBR	RNU6	N	1	1	Clinical symptoms; ECG changes; increase cTnI, CK and CK-MB	Within 24 h of symptoms onset
Agiannitopoulos et al. (2018)	TaqMan	RNU24	N	2	2	Ischemic chest pain; ST segment elevation or depression; pathological Q wave; rise of cardiac biomarkers	Within 24 h of onset of chest pain
Liu et al. (2018)	SYBR	n/a	N	4	4	ACC guideline	Within 2–4 h after admission
Wang et al. (2019)	n/a	Cel-miR-39	N	6	2	Ischemic symptoms; ST segment-T wave changes or new LBBB; pathological Q wave; coronary angiography	Within 4 h of onset of chest pain/dyspnea
Yi and An (2018)	SYBR	RNU6	N	1	1	Coronary angiography; increase cTns and CK-MB	NA
Zhang et al. (2018)	SYBR	n/a	N	1	1	ST segment elevation; coronary angiography	Within 6 h after the onset of symptoms
Bukauskas et al. (2019)	SYBR	Cel-miR-39	N	3	3	2015 ESC Guidelines for the management of ACS	the first 24 h of admission
Li H. et al. (2019)	SYBR	Cel-miR-39	N	3	3	ACC/AHA guideline	With chest pain for 10 h
Li P. et al. (2019)	SYBR	RNU6	N	4	4	Ischemic symptoms; ST segment elevation or pathological Q wave; increase cTnI	With chest pain for 3 h
Xue et al. (2019a)	SYBR	Cel-miR-39	N	3	3	2012 ESC/AHA/ACC guidelines	Within 4 h of onset of chest pain
Xue et al. (2019b)	SYBR	Cel-miR-39	N	6	3	2012 ESC/AHA/ACC guidelines	Chest pain onset <4 h duration

*SYBR, SYBR Green; RNU6, small nuclear RNA U6; STEMI, ST-elevation myocardial infarction; NSTEMI, non ST-elevation myocardial infarction; CK, creatine kinase; CK-MB, creatine kinase-MB. Cardiac troponin I/T, cTnI/T. ECG, electrocardiogram*.

*(n): the number of dysregulated miRNAs*.

a *Based upon miRNA screening, or based upon literature review or prior work (see text)*.

*Y or N, Yes or No; NA: not available*.

**Table 3:** Significantly dysregulated miRNAs of patients with AMI as compared with controls.

**miRNAs**	**References**	**Expression**	**Area under the curve (AUC)**	**Sensitivity**	**Specificity**
**Single miRNA**					
miRNA-1	Ai et al., 2010 Corsten et al., 2010 Cheng et al., 2010 D'Alessandra et al., 2010 Wang et al., 2010 Gidlöf et al., 2011 Li Y. Q. et al., 2013 Long et al., 2012a Olivieri et al., 2013 Li C. et al., 2013 Gidlöf et al., 2013 Li L. M. et al., 2014 Liu et al., 2015 Liu et al., 2018	Upregulated	0.774 —^a^ — — 0.847 0.979 0.827 0.92 — — 0.59 0.854 0.81 0.773	70.0% 90.0% — — 78.0% 88.9% 80.0% 93.0% — 78.0% 55.0% 80.0% 70.0% 75.7%	75.0% 95.0% — — 80.0% 100% 90.0% 90.0% — 86.0% 65.0% 90.0% 90.0% 53.5%
miRNA-17-5p	Xue et al., 2019a	Upregulated	0.857	85.2%	85.7%
miRNA-19b-3p	Wang et al., 2016 Wang et al., 2019	Upregulated	0.821 0.667	72.2% 66.1%	66.7% 61.3%
miRNA-21 miRNA-21-5p	Zile et al., 2011 Olivieri et al., 2013 Zhang et al., 2016 Wang et al., 2014	Upregulated	— — 0.892 0.949	— — 78.6% —	— — 100% —
miRNA-23b	Zhang et al., 2018	Upregulated	0.809	88.1%	60.3%
miRNA-26a-1	Xue et al., 2019b	Upregulated	0.965	100%	85.2%
miRNA-30a	Long et al., 2012b	Upregulated	0.88	88.3%	82.5%
miRNA-122-5p	Yao et al., 2015 Wang et al., 2019	Upregulated	0.855 0.626	100% 63.8%	60.2% 73.9%
miRNA-124	Guo et al., 2017	Upregulated	0.86	52.0%	91.0%
miRNA-126 miRNA-126-5p	Long et al., 2012a Xue et al., 2019a	Upregulated	0.86 0.802	75.1% 100%	83.1% 61.9%
miRNA-133 miRNA-133a miRNA-133b	Wang et al., 2011 Peng et al., 2014 Liu et al., 2018 Corsten et al., 2010 D'Alessandra et al., 2010 Wang et al., 2010 Gidlöf et al., 2011 Li Y. Q. et al., 2013 Olivieri et al., 2013 Wang et al., 2013 Ji et al., 2015 Ke-Gang et al., 2016 D'Alessandra et al., 2010 Ji et al., 2015	Upregulated	0.89 0.912 0.928 — — 0.867 0.859 0.947 — — 0.787 0.667 — 0.823	98.0% 81.1% 71.0% — — 87.3% 99.6% 82.7% — — 85.4% 61% — 66.5%	73.0% 91.2% 96.5% — — 84.9% 63.6% 100% — — 99.8% 68% — 100%
miRNA-134 miRNA-134-5p	He et al., 2014 Li C. et al., 2013 Wang et al., 2016 Wang et al., 2019	Upregulated	0.818 0.657 0.827 0.702	79.4% 53.6% 77.8% 70.6%	77.1% 78.1% 77.8% 79.1%
miRNA-145-3p	Xue et al., 2019a	Upregulated	0.720	81.8%	61.9%
miRNA-146a	Xue et al., 2019b	Upregulated	0.911	100%	66.7%
miRNA-150-3p miRNA-150	Hsu et al., 2014 Li H. et al., 2019 Zhang R. et al., 2015	Upregulated	0.715 0.904 0.678	71.3% 88.2% 80.6%	70.1% 75.9% 49.8%
miRNA-181a	Zhu et al., 2016	Upregulated	0.834	81.5%	81.8%
miRNA-186 miRNA-186-5p	Li C. et al., 2013 Wang et al., 2016 Wang et al., 2019	Upregulated	0.715 0.824 0.692	77.8% 77.8% 56.9%	60.9% 77.8% 84.3%
miRNA-195	Long et al., 2012b	Upregulated	0.89	100%	44.7%
miRNA-199a-1	Xue et al., 2019b	Upregulated	0.855	96.8%	66.7%
miRNA-208b miRNA-208a miRNA-208	Corsten et al., 2010 Gidlöf et al., 2011 Devaux et al., 2012 Li Y. Q. et al., 2013 Gidlöf et al., 2013 Devaux et al., 2015 Li et al., 2015 Agiannitopoulos et al., 2018 Wang et al., 2010 Xiao et al., 2014 Białek et al. (2015) Li C. et al., 2013 Han et al., 2015 Liu et al., 2015 Liu et al., 2018 Li P. et al., 2019	Upregulated	0.944 1.0 0.90 0.89 0.82 0.76 0.674 0.999 0.965 — — 0.778 — 0.72 0.9940 0.868	90.6% 100% 79.5% 82.4% 79.0% 64.7% 59.8% 98% 90.9% — — 75.8% — 65.0% 90.0% 70.0%	94.1% 100% 99.8% 99.8% 70.0% 80.2% 73.6% 100% 100% — — 73.1% — 90.0% 100% 97.5%
miRNA-210	Shalaby et al., 2016	Upregulated	0.90	83.3%	100%
miRNA-223	Li C. et al., 2013	Upregulated	0.741	77.8%	68.3%
miRNA-302b	Yang et al., 2017	Upregulated	0.95	88.2%	93.3%
miRNA-328	He et al., 2014	Upregulated	0.887	86.3%	74.6%
miRNA-361-5p	Wang et al., 2014	Upregulated	0.881	—	—
miRNA-423-5p	Olivieri et al., 2013	Upregulated	—	—	—
miRNA-486-3p miRNA-486	Hsu et al., 2014 Zhang R. et al., 2015	Upregulated	0.629 0.731	38.8% 56.5%	84.1% 86.5%
miRNA-494	Li P. et al., 2019	Upregulated	0.839	79.6%	82.1%
miRNA-497	Li Z. et al., 2014	Upregulated	0.88	82%	94%
miRNA-499 miRNA-499-5p miRNA-499a	Corsten et al., 2010 Adachi et al., 2010 Wang et al., 2010 Devaux et al., 2012 Li Y. Q. et al., 2013 Li C. et al., 2013 Xiao et al., 2014 Chen et al., 2015 Devaux et al., 2015 Liu et al., 2015 Zhang L. et al., 2015 Zhao et al., 2015 Shalaby et al., 2016 Agiannitopoulos et al., 2018 Liu et al., 2018 Li P. et al., 2019 D'Alessandra et al., 2010 Gidlöf et al., 2011 Olivieri et al., 2013 Gidlöf et al., 2013 Fawzy et al., 2018	Upregulated	0.918 — 0.822 0.98 0.884 0.755 — — 0.65 0.88 0.86 0.915 0.97 0.999 0.995 0.852 — 0.989 0.88 0.79 0.953	84.5% — 60.0% 95.0% 80.0% 80.0% — — 35.7% 82.0% 80% 86.37% 93.4% 98% 98.4% 71.5% — 87.7% 88.0% 64.0% 97.2%	93.9% — 90.3% 100% 94.0% 93.0% — — 90.3% 94.0% 80.28% 93.47% 100% 100% 100% 89.3% — 100% 75.0% 90.0% 75%
miRNA-663b	Meder et al., 2011 Peng et al., 2014	Upregulated	0.94 0.611	90% 72.4%	95% 76.5%
miRNA-1291	Peng et al., 2014	Upregulated	0.695	78.4%	89.5%
miRNA-1303	Li H. et al., 2019	Upregulated	0.884	81.2%	89.3%
miRNA-22-5p	Wang et al., 2019	Downregulated	0.975	96.7%	96.7%
miRNA-23a-3p	Bukauskas et al., 2019	Downregulated	0.806	73.3%	79.6%
miRNA-26a-5p miRNA-26a	Hsu et al., 2014 Li et al., 2015	Downregulated	0.675 0.745	57.8% 73.6%	90.2% 72.4%
miRNA-30d-5p	Bukauskas et al., 2019	Downregulated	0.745	80.9%	64.9%
miRNA-99a	Yang et al., 2016	Downregulated	—	—	—
miRNA-125b	Huang et al., 2014	Downregulated	0.858	—	—
miRNA-126-3p	Hsu et al., 2014	Downregulated	0.694	64.1%	80.0%
miRNA-132-5p	Li H. et al., 2019	Downregulated	0.886	85.3%	74.1%
miRNA-145	Gao et al., 2015	Downregulated	—	—	—
miRNA-146a-5p	Bukauskas et al., 2019	Downregulated	0.800	84.5%	70.4%
miRNA-191-5p miRNA-191	Hsu et al., 2014 Li et al., 2015	Downregulated	0.652 0.669	48.6% 62.1%	93.8% 69.0%
miRNA-214	Lu et al., 2013	Downregulated	—	—	—
miRNA-320b	Huang et al., 2014	Downregulated	0.866	—	—
miRNA-375	D'Alessandra et al., 2010 Wang et al., 2019	Downregulated	— 0.510	— 92.6%	— 33.1%
miRNA-379	Yi and An, 2018	Downregulated	0.751	—	—
miRNA-519-5p	Wang et al., 2014	Downregulated	0.798	—	—
miRNA-let-7d	Long et al., 2012b	Downregulated	0.86	64.7%	100%
**Two miRNA panels**					
miRNA-191-5p,-486-3p	Hsu et al., 2014	Upregulated and downregulated^b^	0.867	83.87%	83.33%
miRNA-486-3p,-126-3p	Hsu et al., 2014	Upregulated	0.849	61.29%	93.55%
miRNA-126-3p,-150-3p	Hsu et al., 2014	Upregulated	0.843	93.55%	64.52%
miRNA-486-3p,-26a-5p	Hsu et al., 2014	Upregulated and downregulated	0.821	87.10%	64.52%
miRNA-26a-5p,-150-3p	Hsu et al., 2014	Upregulated and downregulated	0.821	83.87%	70.97%
miRNA-191-5p,-150-3p	Hsu et al., 2014	Upregulated and downregulated	0.789	77.42%	77.42%
miRNA-150,-486	Zhang R. et al., 2015	Upregulated	0.771	72.6%	72.1%
miRNA-499,-210	Shalaby et al., 2016	Upregulated	0.98	97.9%	100%
miRNA-22-5p,-122-5p	Wang et al., 2019	Upregulated and downregulated	0.976	98.4%	96.2%
**Three miRNA panels**					
miRNA-30a,-195, let-7d	Long et al., 2012b	Upregulated and downregulated	0.930	94%	90%
miRNA-21-5p,-361-5p,-519-5p	Wang et al., 2014	Upregulated and downregulated	0.989	93.9%	100%
miRNA-19b-3p,-134-5p,-186-5p	Wang et al., 2016	Upregulated	0.898	88.9%	77.8%
miRNA-22-5p,-150-3p,-132-5p	Li H. et al., 2019	Upregulated and downregulated	0.942	91.2%	87.0%
miRNA-17-5p,-126-5p,-145-3p	Xue et al., 2019a	Upregulated	0.857	84.0%	85.7%
miRNA-26a-1,-146a,-199a-1	Xue et al., 2019b	Upregulated	0.913	97.8%	71.6%
**Panels with** **≥** **4 miRNAs**					
miRNA-1,-134,-186,-208,-223,-499	Li C. et al., 2013	Upregulated	0.811	55.3%	90.1%

*AMI, acute myocardial infarction; miRNA, microRNA*.

a *Not reported*.

b *Upregulated and downregulated: where the individual miRNAs were either upregulated or downregulated in AMI compared with control groups*.

### Sensitivity, Specificity, and Area Under the Curve

Among the included studies, the most common methods of assessing the diagnostic accuracy of dysregulated miRNAs were AUC and sensitivity and specificity, as determined from ROC curves. In the identified dysregulated miRNAs, AUC values ranged from 0.510 to 1.0, sensitivity ranged from 48.6% to 100%, and specificity from 33.1% to 100%. In the single identified miRNAs, the highest AUC sensitivity and specificity combination was reported for miRNA-208b (AUC 1.0, sensitivity 100%, specificity 100%) (Gidlöf et al., 2011). In the panel of two miRNAs, the highest value combination was reposted for miRNA-499 and miRNA-210 (AUC 0.98, sensitivity 97.9%, specificity 100%) (Shalaby et al., 2016). In the panel of three miRNAs, the highest value combination was reported for miRNA-21-5p, miRNA-361-5p, and miRNA-519-5p (AUC 0.989, sensitivity 93.9%, specificity 100%) (Wang et al., 2014). In the panel of ≥ 4 miRNAs, there was one study about AUC 0.811, which had sensitivity 55.3% and specificity 90.1% (Li C. et al., 2013).

### Meta-Analysis Outcomes of Diagnostic Accuracy of miRNAs in AMI

#### The Quality Assessment of Included Studies

**Figure 2:**
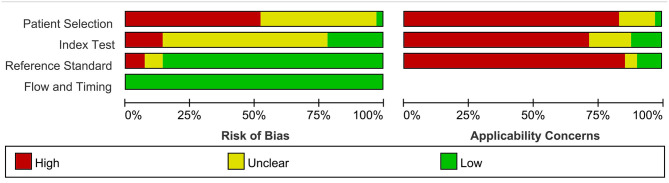
Bar graphs of the methodological quality assessment. Each risk-of-bias and applicability item is presented as percentages across included studies, which indicates the proportion of different levels for each item.

The pooled result of the meta-analysis for the diagnostic accuracy of blood-based miRNAs in AMI was pooled from 42 studies (Ai et al., 2010; Corsten et al., 2010; Wang et al., 2010, 2011, 2014, 2016, 2019; Gidlöf et al., 2011, 2013; Meder et al., 2011; Devaux et al., 2012, 2015; Long et al., 2012a,b; Li C. et al., 2013; Li Y. Q. et al., 2013; Olivieri et al., 2013; He et al., 2014; Hsu et al., 2014; Li L. M. et al., 2014; Li Z. et al., 2014; Peng et al., 2014; Ji et al., 2015; Li et al., 2015; Liu et al., 2015, 2018; Yao et al., 2015; Zhang L. et al., 2015; Zhang R. et al., 2015; Ke-Gang et al., 2016; Shalaby et al., 2016; Yang et al., 2016; Zhang et al., 2016, 2018; Zhu et al., 2016; Guo et al., 2017; Agiannitopoulos et al., 2018; Fawzy et al., 2018; Bukauskas et al., 2019; Li H. et al., 2019; Li P. et al., 2019; Xue et al., 2019a,b). Quality assessment results of the studies reporting on miRNAs included in the meta-analysis using the QUADAS-2 evaluation tool are shown in Supplementary Figure 1. Results are presented as percentages across the studies (Figure 2).

#### Total miRNAs

Dysregulated miRNAs in AMI patients compared with controls, the SROC curve with AUC, sensitivity, specificity, PLR, NLR, and DOR for miRNAs were included in our meta-analysis (Table 4). A random effect model was used for the meta-analysis due to significant heterogeneity (all *I*^2^ > 50%). Forty-four individual miRNAs were identified in 43 studies (Ai et al., 2010; Corsten et al., 2010; Wang et al., 2010, 2011, 2014, 2016, 2019; Gidlöf et al., 2011, 2013; Meder et al., 2011; Devaux et al., 2012, 2015; Long et al., 2012a,b; Li C. et al., 2013; Li Y. Q. et al., 2013; Olivieri et al., 2013; He et al., 2014; Hsu et al., 2014; Li L. M. et al., 2014; Li Z. et al., 2014; Peng et al., 2014; Ji et al., 2015; Li et al., 2015; Liu et al., 2015, 2018; Yao et al., 2015; Zhang L. et al., 2015; Zhang R. et al., 2015; Ke-Gang et al., 2016; Shalaby et al., 2016; Yang et al., 2016; Zhang et al., 2016, 2018; Zhu et al., 2016; Guo et al., 2017; Agiannitopoulos et al., 2018; Fawzy et al., 2018; Bukauskas et al., 2019; Li H. et al., 2019; Li P. et al., 2019; Xue et al., 2019a,b). The sensitivity, specificity, and the corresponding SROC value with 95% CIs (95% confidential intervals) of the total miRNAs in the diagnostic of AMI were 0.82 (95% CI: 0.79–0.85), 0.87 (95 %CI: 0.84–0.90), and 0.91 (95% CI: 0.88–0.93), respectively (Supplementary Figures 2A,B, 3A). The Deeks' funnel plot asymmetry test suggested a potential for publication bias in the total miRNAs (*p-*value = 0.00, Supplementary Figure 3B).

**Table 4:** The overall and subgroups meta-analysis results for comparison of diagnostic value of miRNAs.

**Comparisons (*n*, study)**	**AUC (95% CI)**	**Sensitivity (95% CI)**	**Specificity (95% CI)**	**PLR (95% CI)**	**NLR (95% CI)**	**DOR (95% CI)**
**POOLED SINGLE miRNAs (*****n*** **=** **102)**						
**Total miRNAs**	0.91 (0.88–0.93)	0.82 (0.79–0.85)	0.87 (0.84–0.90)	6.27 (4.97–7.90)	0.21 (0.18–0.24)	30.40 (21.60–42.77)
**Multiple combinations**						
Two miRNAs panel (*n* = 9)	0.92 (0.90–0.94)	0.88 (0.77–0.94)	0.84 (0.72–0.91)	5.40 (2.84–10.26)	0.15 (0.07–0.30)	37.00 (10.52–130.16)
Three miRNAs panel (*n* = 6)	0.92 (0.89–0.94)	0.91 (0.85–0.94)	0.87 (0.77–0.92)	6.70 (3.83–11.72)	0.11 (0.07–0.18)	62.24 (27.40–141.38)
**SUBGROUP ANALYSIS**						
**Type of blood sample**						
Plasma (*n* = 75)	0.92 (0.89–0.94)	0.84 (0.80–0.87)	0.87 (0.82–0.90)	6.22 (4.68–8.27)	0.19 (0.15–0.23)	32.97 (21.48–50.61)
Serum (*n* = 26)	0.89 (0.86–0.92)	0.78 (0.72–0.82)	0.87 (0.82–0.91)	6.13 (4.23–8.89)	0.26 (0.20–0.33)	23.88 (14.15–40.28)
**Type of miRNA detection method**						
SYBR green (*n* = 62)	0.92 (0.89–0.94)	0.84 (0.80–0.87)	0.87 (0.83–0.90)	6.33 (4.77–8.40)	0.19 (0.15–0.24)	33.83 (22.46–50.94)
TaqMan (*n* = 30)	0.90 (0.87–0.92)	0.80 (0.74–0.85)	0.88 (0.82–0.93)	6.82 (4.27–10.88)	0.23 (0.17–0.30)	30.15 (15.03–60.45)
**Type of miRNA reference**						
RNU (*n* = 27)	0.93 (0.91–0.95)	0.86 (0.80–0.91)	0.89 (0.81–0.94)	8.08 (4.42–14.78)	0.16 (0.11–0.23)	51.49 (22.59–117.35)
Cel-miRNA (*n* = 50)	0.90 (0.87–0.92)	0.82 (0.78–0.85)	0.85 (0.81–0.89)	5.55 (4.22–7.29)	0.21 (0.17–0.26)	26.36 (17.70–39.25)
**Included studies size**						
Sample size ≥ 100 (*n* = 51)	0.89 (0.86–0.91)	0.79 (0.75–0.83)	0.86 (0.81–0.90)	5.82 (4.04–8.37)	0.24 (0.19–0.31)	24.00 (13.91–41.42)
Sample size <100 (*n* = 51)	0.93 (0.90–0.95)	0.85 (0.81–0.89)	0.87 (0.83–0.90)	6.65 (5.03–8.78)	0.17 (0.13–0.21)	39.75 (27.00–58.53)
**Different population**						
Caucasian (*n* = 21)	0.95 (0.93–0.97)	0.86 (0.78–0.91)	0.94 (0.86–0.97)	14.15 (5.89–34.00)	0.15 (0.09–0.24)	95.63 (28.08–325.74)
East Asian (*n* = 78)	0.89 (0.86–0.91)	0.80 (0.77–0.83)	0.84 (0.81–0.87)	5.12 (4.13–6.35)	0.23 (0.20–0.28)	21.88 (15.97–29.99)
**Type of different miRNAs**						
miR-1 (*n* = 11)	0.88 (0.85–0.90)	0.78 (0.71–0.84)	0.86 (0.77–0.91)	5.41 (3.18–9.19)	0.26 (0.18–0.36)	21.07 (9.17–48.38)
miR-133 a/b (*n* = 9)	0.94 (0.92–0.96)	0.85 (0.72–0.92)	0.92 (0.78–0.98)	10.79 (3.63–32.09)	0.17 (0.09–0.31)	64.18 (19.03–216.49)
miR-133a (*n* = 5)	0.93 (0.91–0.95)	0.85 (0.69–0.94)	0.92 (0.61–0.99)	10.63 (1.69–66.96)	0.16 (0.07–0.36)	66.15 (7.41–590.92)
miR-208 a/b (*n* = 13)	0.94 (0.91–0.95)	0.83 (0.74–0.89)	0.98 (0.88–0.99)	35.45 (5.90–212.88)	0.18 (0.11–0.28)	201.13 (24.36–1660.71)
miR-208b (*n* = 7)	0.91 (0.88–0.93)	0.80 (0.69–0.88)	0.96 (0.77–0.99)	19.30 (2.78–134.22)	0.21 (0.12–0.35)	92.61 (9.07–945.66)
miR-499 (*n* = 17)	0.96 (0.94–0.97)	0.85 (0.77–0.91)	0.95 (0.89–0.98)	16.27 (7.31–36.22)	0.16 (0.10–0.26)	103.54 (31.08–345.01)

*miR, microRNA. AUC, the area under cure in the overall summary receiver operator characteristic (SROC) curve. CI, confidence interval; PLR, positive likelihood ratio. NLR, negative likelihood ratio; DOR, diagnostic odds ratio; n, the number of included studies*.

#### Panels of Multiple miRNAs

**Figure 3:**
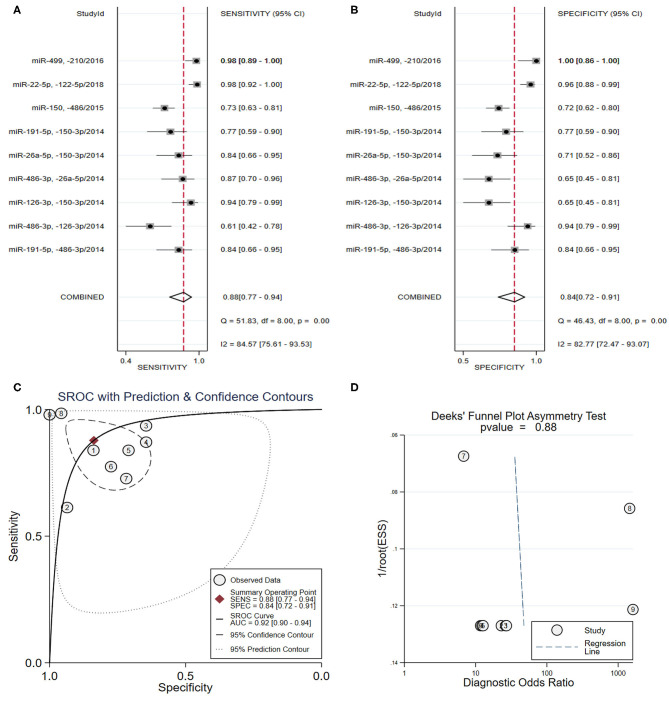
The sensitivity, specificity, summary receiver operator characteristic (SROC) curve with area under curve (AUC), and funnel graph of the combination of two miRNAs in the diagnosis of acute myocardial infarction. **(A)** Sensitivity. **(B)** Specificity. **(C)** SROC curve with AUC. **(D)** Funnel graph.

As shown in Table 3, four studies (Hsu et al., 2014; Zhang R. et al., 2015; Shalaby et al., 2016; Wang et al., 2019) focused on the diagnostic value of a panel of two types of miRNAs, and the pooled sensitivity (Figure 3A) and specificity (Figure 3B) estimates were 0.88 (95% CI: 0.77–0.94) and 0.84 (95% CI: 0.72–0.91), respectively. The area under the SROC curve (Figure 3C) was 0.92 (95% CI: 0.90–0.94). The Deeks' test (Figure 3D) was performed to evaluate publication bias, and results suggested a low probability of publication bias.

**Figure 4:**
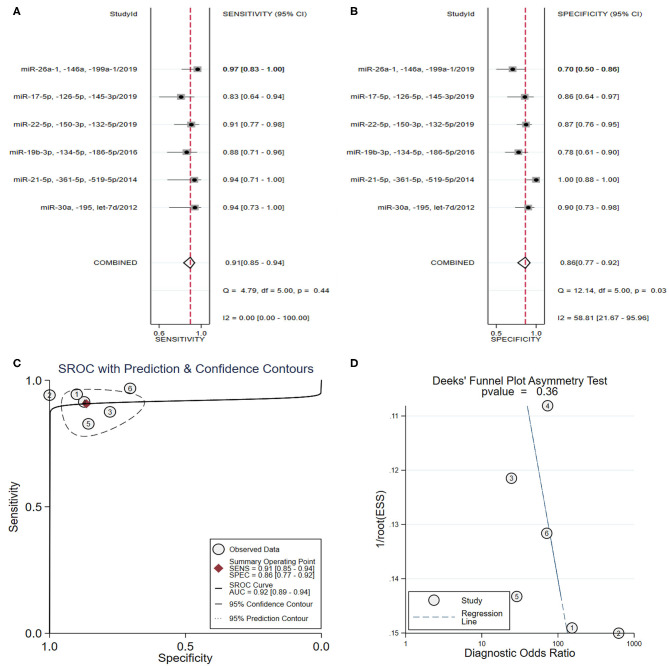
The sensitivity, specificity, summary receiver operator characteristic (SROC) curve with area under curve (AUC), and funnel graph of the combination of three miRNAs in the diagnosis of acute myocardial infarction. **(A)** Sensitivity. **(B)** Specificity. **(C)** SROC curve with AUC. **(D)** Funnel graph.

From the analysis of a panel of three types of miRNAs in six studies (Long et al., 2012b; Wang et al., 2014, 2016; Ke-Gang et al., 2016; Li H. et al., 2019; Xue et al., 2019a,b) (Table 3), the pooled sensitivity (Figure 4A) and specificity (Figure 4B) estimates were 0.91 (95% CI: 0.85–0.94) and 0.87 (95% CI: 0.77–0.92), respectively. The area under the SROC curve (Figure 4C) was 0.92 (95% CI: 0.89–0.94). The Deeks' test was performed and suggested that publication bias likely had a low effect on the summary estimates (Figure 4D).

#### Sensitivity Analyses

Sensitivity analyses were performed on the included studies according to the following factors: (1) Type of patient blood sample (plasma vs. serum): the SROC values were 0.92 vs. 0.89, the pooled sensitivity and specificity were 0.84 vs. 0.78 and 0.87 vs. 0.87, respectively; (2) Type of miRNA detection method (SYBR green vs TaqMan): the SROC values were 0.92 vs. 0.90, the pooled sensitivity and specificity were 0.84 vs. 0.80 and 0.87 vs. 0.88, respectively; (3) Type of miRNA reference used for normalization (RNU or Cel-miRNA): the SROC values were 0.93 vs. 0.90, the pooled sensitivity and specificity were 0.86 vs. 0.82 and 0.89 vs. 0.85, respectively; (4) different study sizes (sample size ≥ 100 vs. sample size <100): the SROC values were 0.89 vs 0.93, the pooled sensitivity and specificity were 0.79 vs. 0.85 and 0.86 vs. 0.87, respectively; (5) Different populations (Caucasian vs. East Asian): the SROC values were 0.95 vs. 0.89, the pooled sensitivity and specificity were 0.86 vs. 0.80 and 0.94 vs. 0.84, respectively. A summary of the sensitivity analysis results is shown in Table 4. According to compared diagnostic values from each of the above groups, no major improvement or differences were found in the diagnostic accuracy values.

### Meta-Analysis by Types of miRNAs

#### miRNA-1

**Figure 5:**
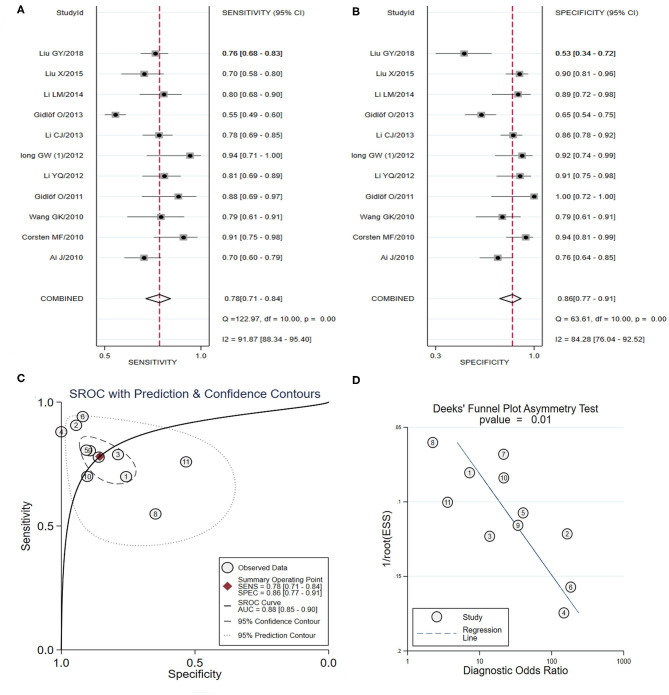
The sensitivity, specificity, summary receiver operator characteristic (SROC) curve with area under curve (AUC), and funnel graph of miRNA-1 in the diagnosis of acute myocardial infarction. **(A)** Sensitivity. **(B)** Specificity. **(C)** SROC curve with AUC. **(D)** Funnel graph.

In total, 11 studies identifying the diagnostic value of miRNA-1 in AMI were included in the meta-analysis (Ai et al., 2010; Corsten et al., 2010; Wang et al., 2010; Gidlöf et al., 2011, 2013; Long et al., 2012a; Li C. et al., 2013; Li Y. Q. et al., 2013; Li L. M. et al., 2014; Liu et al., 2015, 2018) (Table 3). As shown in Figures 5A,B, the pooled sensitivity and specificity estimates were 0.78 (95% CI: 0.71–0.84) and 0.86 (95% CI: 0.77–0.91), respectively. The area under the SROC curve for miRNA-1 was 0.88 (95% CI: 0.85–0.90) (Figure 5C). The pooled PLR was 5.41 (95% CI: 3.18–9.19), and the pooled NLR was 0.26 (95% CI: 0.18–0.36). The DOR was 21.07 (95% CI: 9.17–48.38). Additionally, the Deeks' test suggested that publication bias may have some effect on the summary estimates (*p-*value = 0.01) (Figure 5D). Representative results from the above analyses are shown in Table 4.

#### miRNA-133

**Figure 6:**
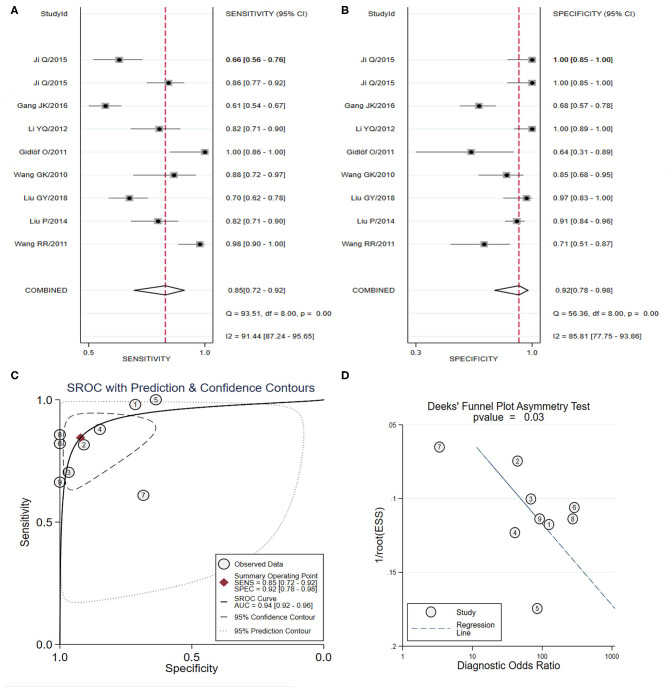
The sensitivity, specificity, summary receiver operator characteristic (SROC) curve with area under curve (AUC), and funnel graph of the miRNA-133 family in the diagnosis of acute myocardial infarction. **(A)** Sensitivity. **(B)** Specificity. **(C)** SROC curve with AUC. **(D)** Funnel graph.

Nine studies that focused on the diagnostic accuracy of miRNA-133 (including miRNA-133a/b) in AMI were included in the meta-analysis (Corsten et al., 2010; D'Alessandra et al., 2010; Wang et al., 2010, 2011, 2013; Gidlöf et al., 2011; Li Y. Q. et al., 2013; Olivieri et al., 2013; Peng et al., 2014; Ji et al., 2015; Ke-Gang et al., 2016; Liu et al., 2018) (Table 3). As shown in Figures 6A,B, the pooled sensitivity and specificity estimates with 95% CI were 0.85 (95% CI: 0.72–0.92) and 0.92 (95% CI: 0.78–0.98), respectively. The area under the SROC curve for miRNA-133 was 0.94 (95% CI: 0.92–0.96) (Figure 6C). The pooled PLR was 10.79 (95% CI: 3.63–32.09) and the pooled NLR was 0.17 (95% CI: 0.09–0.31). The DOR was 64.18 (95% CI: 19.03–216.49). The Deeks' test suggested a potential publication bias (*p-*value = 0.01) (Figure 6D). Further subgroup analysis was conducted on miRNA-133a in the diagnosis of AMI and the SROC value, the pooled sensitivity, specificity, and DOR in the five studies (Wang et al., 2010; Gidlöf et al., 2011; Li Y. Q. et al., 2013; Ji et al., 2015; Ke-Gang et al., 2016) were 0.93 (95% CI: 0.91–0.95), 0.85 (95% CI: 0.69–0.94), 0.92 (95% CI: 0.61–0.99), and 66.15 (95% CI: 7.41–590.92). Representative results from the above analyses are shown in Table 4.

#### miRNA-208

**Figure 7:**
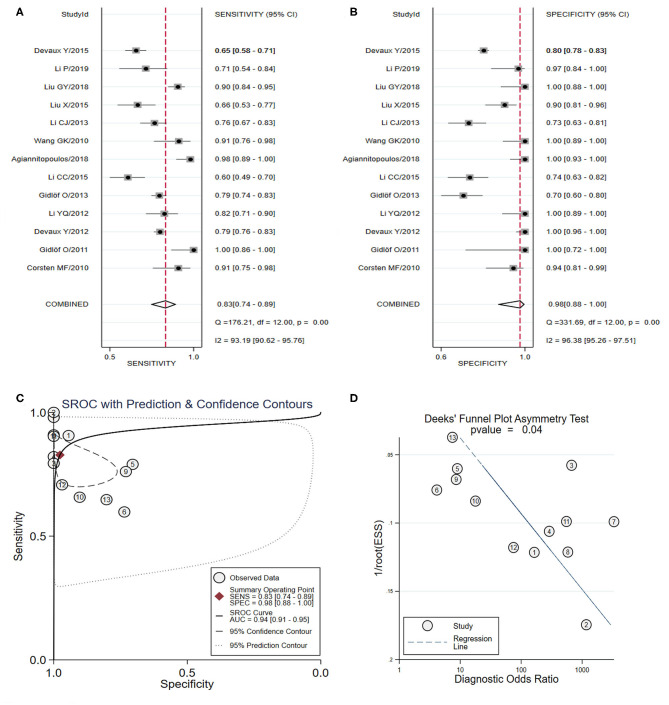
The sensitivity, specificity, summary receiver operator characteristic (SROC) curve with area under curve (AUC), and funnel graph of the miRNA-208 family in the diagnosis of acute myocardial infarction. **(A)** Sensitivity. **(B)** Specificity. **(C)** SROC curve with AUC. **(D)** Funnel graph.

Thirteen studies evaluated the diagnostic value of miRNA-208 (including miRNA-208a/b) in AMI and were included in the meta-analysis (Corsten et al., 2010; Wang et al., 2010; Gidlöf et al., 2011, 2013; Devaux et al., 2012, 2015; Li C. et al., 2013; Li Y. Q. et al., 2013; Li et al., 2015; Liu et al., 2015, 2018; Agiannitopoulos et al., 2018; Li P. et al., 2019) (Table 3). As shown in Figures 7A,B, the pooled sensitivity and specificity estimates with 95% CI were 0.83 (95% CI: 0.74–0.89) and 0.98 (95% CI: 0.88–0.99), respectively. The area under the SROC curve for miRNA-208 was 0.94 (95% CI: 0.91–0.95) (Figure 7C). The pooled PLR was 35.45 (95% CI: 5.90–212.88), and the pooled NLR was 0.18 (95% CI: 0.11–0.28). The DOR was 201.13 (95% CI: 24.36–1660.71). The Deeks' test suggested a possible publication bias (*p-*value = 0.04) (Figure 7D). In the subgroup analysis of miRNA-208b in the diagnosis of AMI, the SROC value, the pooled sensitivity, specificity, and DOR in the seven studies (Corsten et al., 2010; Gidlöf et al., 2011, 2013; Devaux et al., 2012, 2015; Li Y. Q. et al., 2013; Li et al., 2015) were 0.91 (95% CI: 0.88–0.93), 0.80 (95% CI: 0.69–0.88), 0.96 (95% CI: 0.77–0.99), 92.61 (95% CI: 9.07–945.66). Representative results from the above analyses are shown in Table 4.

#### miRNA-499

**Figure 8:**
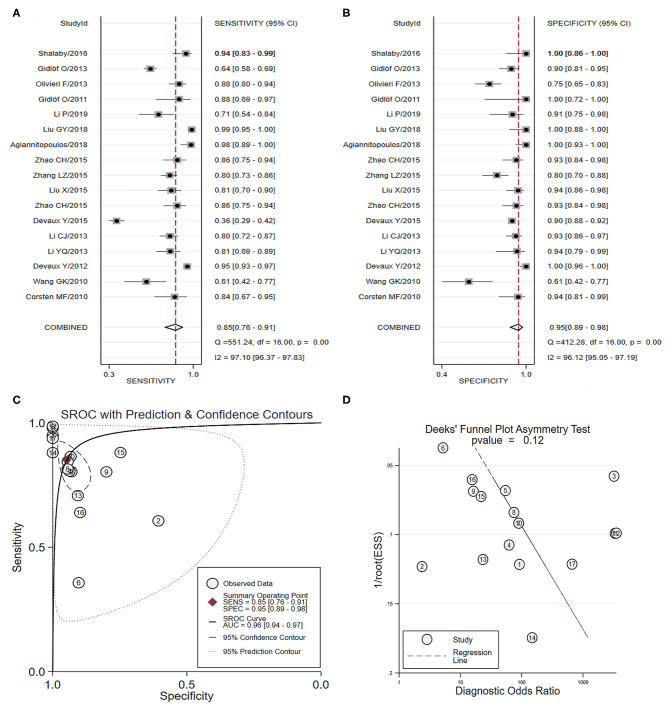
The sensitivity, specificity, summary receiver operator characteristic (SROC) curve with area under curve (AUC), and funnel graph of miRNA-499 in the diagnosis of acute myocardial infarction. **(A)** Sensitivity. **(B)** Specificity. **(C)** SROC curve with AUC. **(D)** Funnel graph.

Seventeen studies involving 3,976 individuals investigated the diagnostic accuracy of miRNA-499 as a novel biomarkers for AMI (Corsten et al., 2010; Wang et al., 2010; Gidlöf et al., 2011, 2013; Devaux et al., 2012, 2015; Li C. et al., 2013; Li Y. Q. et al., 2013; Olivieri et al., 2013; Liu et al., 2015, 2018; Zhang L. et al., 2015; Zhang R. et al., 2015; Shalaby et al., 2016; Agiannitopoulos et al., 2018; Fawzy et al., 2018; Li P. et al., 2019) (Table 3). As shown in Figures 8A,B, the pooled sensitivity and specificity estimates with 95%CI were 0.85 (95% CI: 0.77–0.91) and 0.95 (95% CI: 0.89–0.98), respectively. The area under the SROC curve for miRNA-499 was 0.96 (95% CI: 0.94–0.97) (Figure 8C). The pooled PLR was 16.27 (95% CI: 7.31–36.22), and the pooled NLR was 0.16 (95% CI: 0.10–0.26). The DOR was 103.54 (95% CI: 31.08–345.01). The Deeks' test suggested a low possibility of publication bias (*p-*value = 0.12) (Figure 8D). Representative results from the above analyses are shown in Table 4.

## Discussion

This systematic review and meta-analysis of 58 manuscripts that utilize blood circulating miRNAs (including plasma- or serum-based) in the diagnosis of AMI identified 51 significantly dysregulated miRNAs between AMI cases and controls. Additionally, this review assessed the feasibility of using these miRNAs as novel biomarkers for the diagnosis of AMI patients. Sixteen of the abnormally expressed miRNAs were investigated by more than one study, including thirteen upregulated miRNAs: miRNA-1, miRNA-19b-3p, miRNA-21, miRNA-122-5p, miRNA-126, miRNA-133a/b, miRNA-134, miRNA-150, miRNA-186, miRNA-208a/b, miRNA-486, miRNA-499, and miRNA-663b, and three downregulated miRNAs: miRNA-26a, miRNA-191, and miRNA-375. A further 34 dysregulated miRNAs were only reported by one study. The overall pooled diagnostic data of total miRNAs expression were as follows: SROC curve with AUC: 0.91, sensitivity: 0.82, specificity: 0.87, showing that circulating miRNAs might be suitable for use as potential biomarkers of AMI. Furthermore, the present meta-analysis was conducted via subgroup analyses based on type of miRNAs, including miRNA-1, miRNA-133a/b, miRNA-208a/b, and miRNA-499. MiRNA-499 had the highest diagnostic value (sensitivity: 0.85, specificity: 0.95, SROC curve with AUC: 0.96), followed by miRNA-133a (sensitivity: 0.85, specificity: 0.92, SROC curve with AUC: 0.93), and miRNA-208b had better specificity (0.96) than sensitivity (0.80). These results indicate a relatively high diagnostic accuracy for AMI based on significantly dysregulated miRNAs.

It is well-known that an early, accurate diagnosis and effective revascularization therapy play vital roles in reducing morbidity and mortality in patients with AMI (Yeh et al., 2010). At present, cardiac troponin, creatine kinase-MB (CK-MB), and myoglobin are the most widely used biomarkers in the diagnosis of AMI (de Winter et al., 1995). A good standard for the early diagnosis of AMI is an increase of cTn level (Celik et al., 2011). However, these markers are also likely to be elevated in patients with other diseases, whether or not CAD is also present (French and White, 2004). The detection of cTn has time constraints, as significant levels are reached 4–8 h following the onset of ischemia symptoms. Thus, novel genetic and molecular biomarkers of myocardial damage that have high sensitivity and specificity are still urgently needed.

Significant advances have occurred in the field of cardiovascular disease and miRNAs since their first discovery in the blood (Karakas et al., 2017). A growing number of studies indicate that the abnormal expression of miRNAs plays a critical role due to their various pathological functions in the presence of myocardial infarction (Gurha, 2016; Moghaddam et al., 2019). MiRNAs are steadily present in bodily fluids (including plasma, serum, urine, and saliva) due to protection from RNase via binding to argonaute proteins and the ability to be released from cells via microvesicles, exosomes, or bound to proteins (Meister, 2013). Moreover, recent studies have showed that cTn is more difficult to detect than miRNAs in patients with MI during the earlier acute stage, as it is usually below the cut-off value (Gidlöf et al., 2013; Zhang L. et al., 2015). This suggests a difference between these two types of biomarkers in the physiological process of myocardial infarction. Cardiac troponin releases into the blood during necrosis and during the pathological process of myocardial hypoxia and ischemia (Wu and Ford, 1999). However, miRNAs can be released in response to several forms of cellular stress occurring earlier then cell necrosis such as anoxia, lactic acidosis, and cellular edema (Edeleva and Shcherbata, 2013). Thus, experts may consider the expression levels of dysregulated miRNAs at an earlier stage of AMI, as they might be reliable candidate biomarkers for the diagnosis of AMI (Li C. et al., 2013).

In this study, the summary of single miRNAs showed that miRNA-1, miRNA-133, miRNA-208, and miRNA-499 are potential candidates for the detection of AMI, as they were most frequently detected in the previous studies. Among these individual miRNAs, circulating miRNA-499 might be an effective candidate biomarker of AMI. Some studies have demonstrated that miRNA-499 was specifically expressed in the myocardium and skeletal muscle of mammals (Xue et al., 2019a) and played a critical role in the recovery process following cardiac injury (Hosoda et al., 2011). Based on the present study, miRNA-499 has a higher sensitivity and specificity in identifying patients with AMI, and these results were similar to or better than previous studies (Cheng et al., 2014; Zhao et al., 2019). Previous studies were based on relatively small sample sizes and did not include studies published in recent years. Thus, the results of the present study are more reliable and convincing. Furthermore, we strictly considered the precise setting of specific miRNAs in the diagnosis of AMI and that assessing their diagnostic performance in combination with other biomarkers, especially highly sensitive troponin immunoassays, may be warranted. A study by Olivieri et al. showed a significant correlation between miRNA-499 and cTnT, and that the diagnosis value of this combination was superior to either one alone (Olivieri et al., 2013). However, another study reported that the diagnostic value of miRNA-499 and hs-cTnT combined was not better than either them alone (Devaux et al., 2012). Thus, due to the limited sample sizes of these studies, further research is required.

There are only a small number of high-quality studies with large sample sizes focused on the diagnostic value of miRNAs in AMI. To the best of our knowledge, this study has included the largest sample size of dysregulated miRNAs as novel biomarkers for AMI for summary and evaluation. Further high-quality studies should be performed to acquire more reliable data for use in formulating standard diagnostic criterion and to determine optimal cut-off values. Moreover, due to the different diagnostic values of miRNAs, our study demonstrated that a panel of 2 or 3 miRNAs might be superior for diagnostic accuracy. The combination of 3 miRNAs trended toward a higher sensitivity than others for use as biomarkers in the diagnosis of AMI. Therefore, to further improve the feasibility of clinical diagnosis, future research should explore the most effective combination of multiple miRNAs, especially those confirmed to have a higher utilization value in the single miRNA groups.

Aside from the many studies investigating miRNA profiles in the detection of AMI, researchers should pay closer attention to the search for the technology to detect miRNAs quickly and accurately. Methods of RNA detection tend to be time-consuming, expensive, require sophisticated techniques, and are difficult to implement for urgent testing, especially in some developing countries (Lippi et al., 2013). However, novel detection technologies developed in recent years may provide a solution to these tissues, which would also support the clinical application of miRNAs in the future. Examples of new technologies include isothermal reactions based on cleavage with DNAzyme and signal amplification, which can simultaneously amplify and detect RNA (Zhao et al., 2013). This method is thought to be immune to genomic DNA pollution and has a relatively reliable sensitivity and specificity. We believed that new, precise methods of diagnosis that can detect miRNAs very rapidly and inexpensively need to be continuously improved.

In addition, the heterogeneity of the results of this study were substantial and could not be completely resolved. We found that the sources of heterogeneity included the following: quality of included studies, age, gender proportion, regional and environmental factors, and sampling criteria. Despite the heterogeneity, we believe the results of this study are worthwhile and valuable. Results of the overall and stratified analysis of different subgroups trended toward satisfactory values of miRNAs as novel biomarkers in the diagnosis of AMI. Furthermore, it was remarkable that some publication bias was found in the present study and might imply that the potential negative results were less likely to be published.

Although this study had a satisfactory result regarding the use of miRNAs for AMI detection, conclusions should be drawn cautiously due to several limitations: (1) there was a lack of standardization due to different normalization procedures across the included studies; (2) the exclusion of non-English articles may have caused important studies to be overlooked and publication bias due to significant results being more easily published; (3) the combined analysis of multiple miRNAs for a panel were insufficient, and we were unable to conduct a meta-analysis based on the data from limited studies; and (4) the results may have been affected by the impact of inevitable clinical heterogeneity, including the general condition of the included individuals, effects of medication, and medical history.

## Conclusion

The current systematic review identifies numerous miRNAs associated with AMI and suggested that miRNAs may be used as a potential biomarker for the detection of AMI. For single, stand-alone miRNAs, miRNA-499 had better diagnostic accuracy than other miRNAs. A panel of two or three miRNAs might be superior for diagnostic accuracy. To develop a diagnostic test for AMI diagnosis, we suggest that a panel of miRNAs with high sensitivity and specificity should be tested. For this purpose, large scale, high-quality studies are still required to validate the clinical application of miRNAs for AMI diagnostics, as well as to identify the precise setting of dysregulated miRNAs in patients with AMI.

## Data Availability Statement

Publicly available datasets were analyzed in this study. Datasets are available through the corresponding author upon reasonable request.

## Ethics Statement

All analyses were based on previous published studies; thus no ethical approval or patient consent were required. All previous published studies were approved by Ethics Committee, respectively.

## Author Contributions

HC and CZ designed the study. CZ carried out the statistical analysis and participated in most of the study steps. CZ, RL, JC, KH, and MA prepared the manuscript and assisted in the study processes. YH, JZ, YZ, LW, and RZ assisted in the data collection and helped in the interpretation of the study. All authors read and approved the final manuscript.

## Conflict of Interest

The authors declare that the research was conducted in the absence of any commercial or financial relationships that could be construed as a potential conflict of interest.

## Supplementary Material

Supplementary Figure 1 Methodological quality of studies in the meta-analysis using the Quality Assessment of Diagnostic Accuracy Studies 2 score system, including risk of bias and applicability concerns. The items were scored with “yes,” “no,” or “unsure”.

Supplementary Figure 2 Forest plots of the total miRNAs in the diagnosis of acute myocardial infarction among the studies included in the meta-analysis. **(A)** Sensitivity. **(B)** Specificity.

Supplementary Figure 3 Summary receiver operator characteristic (SROC) curve with area under the curve (AUC) and funnel graph of the total miRNAs in the diagnosis of acute myocardial infarction. **(A)** SROC curve with AUC. **(B)** Funnel graph.
