# Supplementary figures and images for: Value of Blood-Based microRNAs in the Diagnosis of Acute Myocardial Infarction: A Systematic Review and Meta-Analysis
Source: Front Physiol. 2020 Aug 14;11:691. doi: 10.3389/fphys.2020.00691 (PMC7456928; doi:10.3389/fphys.2020.00691)

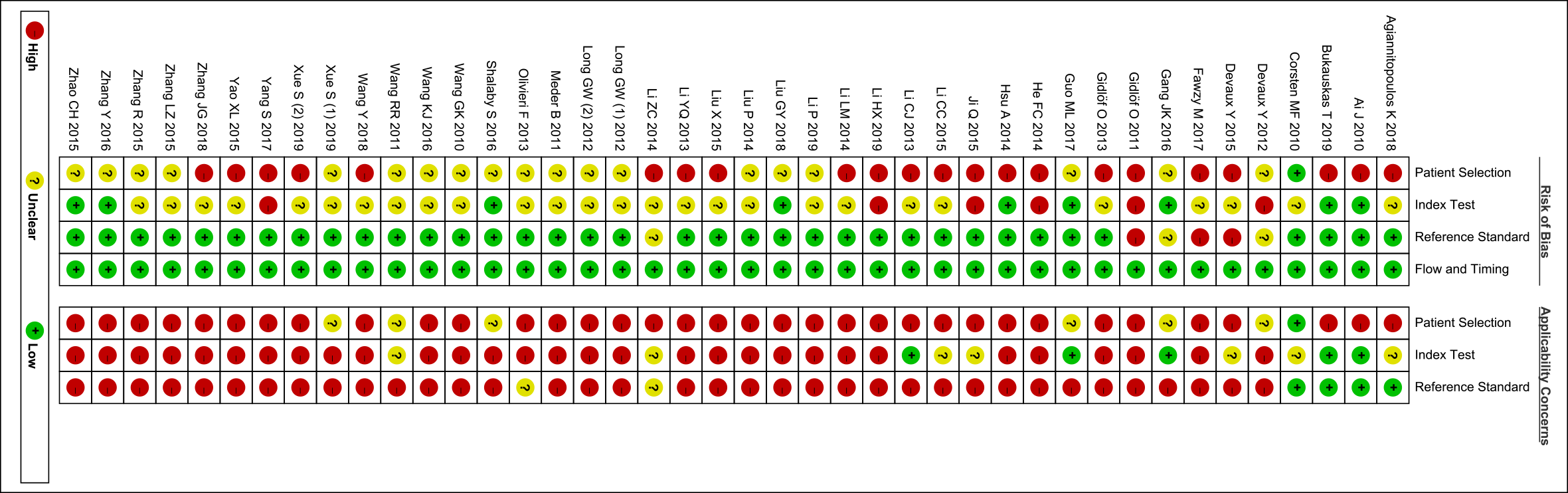

Supplement: Supplementary Figure 1 — Methodological quality of studies in the meta-analysis using the Quality Assessment of Diagnostic Accuracy Studies 2 score system, including risk of bias and applicability concerns. The items were scored with “yes,” “no,” or “unsure”. [file Image_1.TIF]

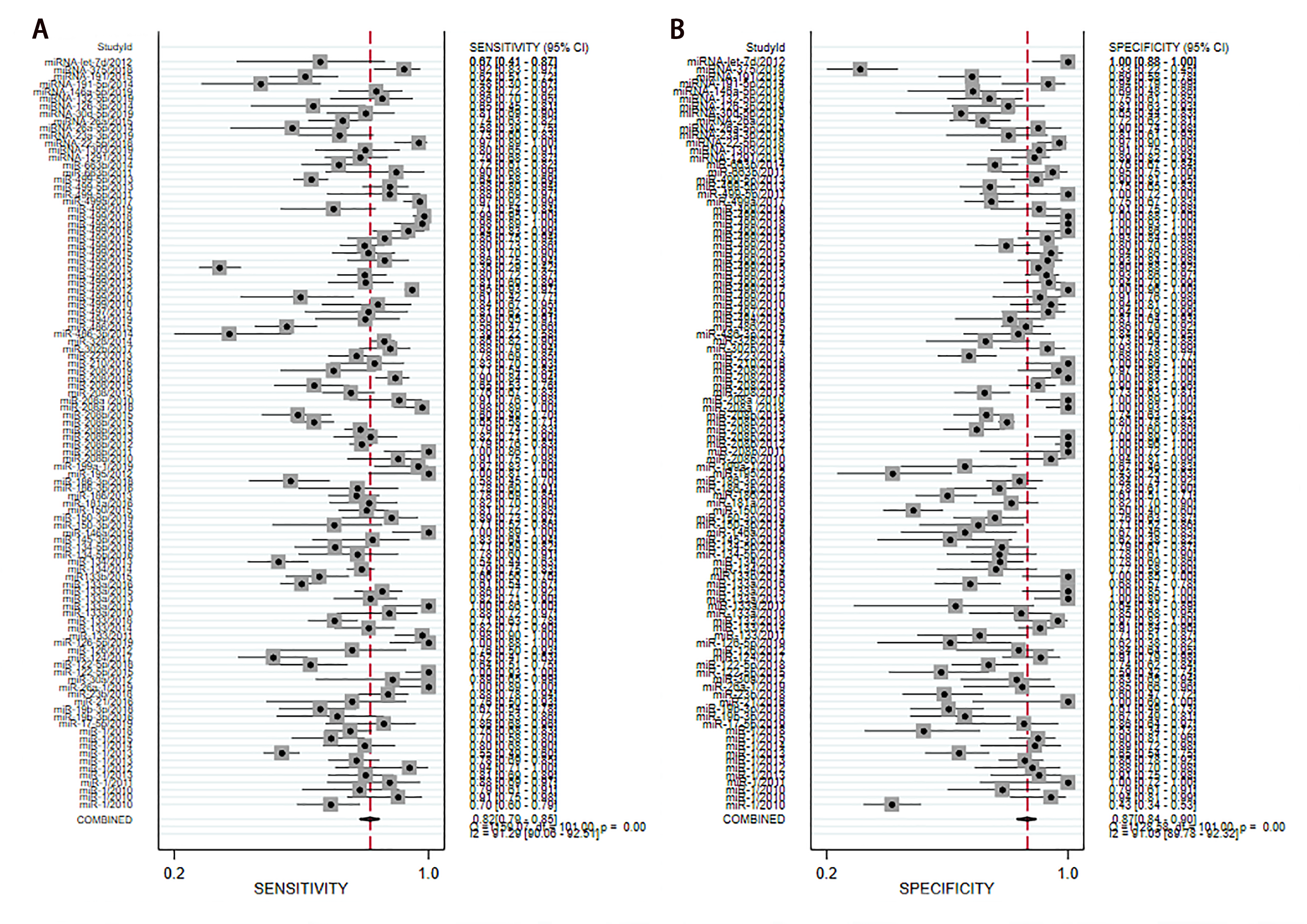

Supplement: Supplementary Figure 2 — Forest plots of the total miRNAs in the diagnosis of acute myocardial infarction among the studies included in the meta-analysis. (A) Sensitivity. (B) Specificity. [file Image_2.TIF]

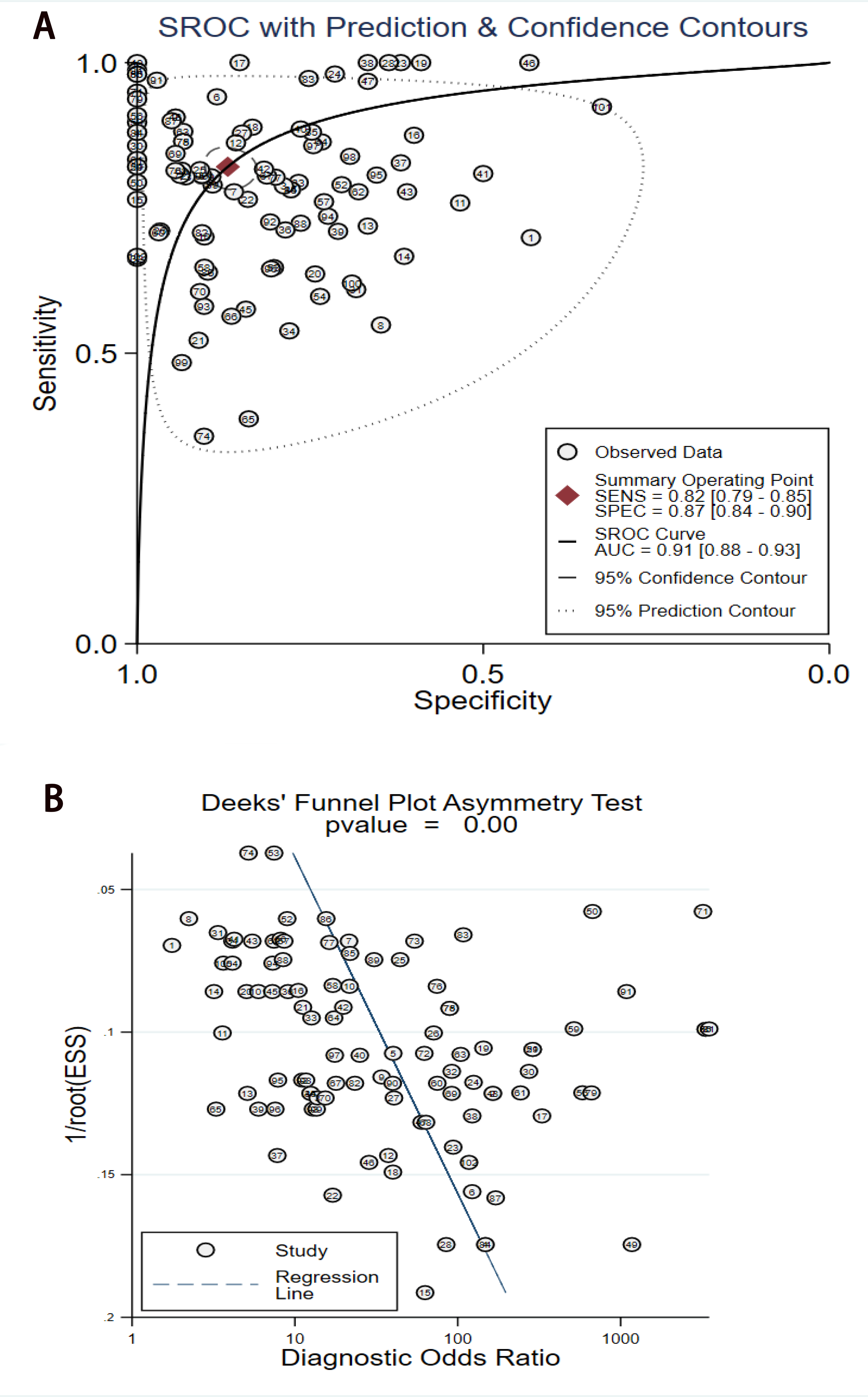

Supplement: Supplementary Figure 3 — Summary receiver operator characteristic (SROC) curve with area under the curve (AUC) and funnel graph of the total miRNAs in the diagnosis of acute myocardial infarction. (A) SROC curve with AUC. (B) Funnel graph. [file Image_3.TIF]
